# Bee Bread as a Functional Product: Phenolic Compounds, Amino Acid, Sugar, and Organic Acid Profiles

**DOI:** 10.3390/foods13050795

**Published:** 2024-03-04

**Authors:** Aksem Aksoy, Sema Sandıkçı Altunatmaz, Filiz Aksu, Nazan Tokatlı Demirok, Kemal Yazıcı, Seydi Yıkmış

**Affiliations:** 1 Department of Food Engineering, Faculty of Engineering Architecture, Kafkas University, 36100 Kars, Türkiye; aksemaksoy@hotmail.com; 2 Food Processing Department, Veterinary Vocational High School, Istanbul University-Cerrahpaşa, Avcılar, 34320 Istanbul, Türkiye; sandikci@iuc.edu.tr (S.S.A.); filiz.aksu@iuc.edu.tr (F.A.); 3 Department of Nutrition and Dietetics, Tekirdağ Namık Kemal University, 59030 Tekirdağ, Türkiye; 4 Department of Plant and Animal Production, Posof Vocational School, Ardahan University, 75800 Ardahan, Türkiye; kemalyazici@ardahan.edu.tr; 5 Department of Food Technology, Tekirdağ Namık Kemal University, 59830 Tekirdağ, Türkiye

**Keywords:** bee bread, bioactive compounds, amino acid, organic acid, chemical analysis

## Abstract

Bee bread (perga) is a natural bee product formed by the fermentation of the pollen collected by bees via lactic acid bacteria and yeasts. This study aims to determine the bioactive compounds, amino acid, sugar, and organic acid profile of bee bread samples collected from the Ardahan province of Türkiye. The highest total phenolic, total flavonoid, and DPPH values in bee bread samples were determined as 18.35 mg GAE/g, 2.82 mg QE/g, and 3.90 mg TEAC/g, respectively. Among phenolic compounds, gallic acid had the highest value at 39.97 µ/g. While all essential amino acids except tryptophan were detected in the samples, aspartic acid was the most dominant, followed by pyrroline and glutamic acid. Among sugars, fructose was seen at the highest level. Succinic acid, among organic acids, had the highest amount at 73.63 mg/g. Finally, all the data were subjected to a principal components analysis (PCA). Bee bread samples were grouped according to the analysis results of the districts they were collected from. This study provides information about the bioactive components and some chemical properties of bee bread, a natural product that has been the subject of recent research. It also contains essential data for future functional food production.

## 1. Introduction

Due to consumers increasing demand for natural products, there is a significant interest in bee products. These products are often researched due to their rich nutritional content and biological activities, making them essential focal points in the food and medicine industries [1,2]. Bee bread, the subject of recent research, is among these products. Th pollens collected by honey bees are mixed with saliva and honey and then stored in honeycomb cells, where they undergo fermentation to become bee bread. Bee bread is a crucial food for bees [3,4]. Also known as fermented bee pollen, this product is formed by the fermentation of bee pollen by lactic acid bacteria and yeasts in the hive. Bee bread contains polyphenols, food components, fatty acids, and minerals [2,4,5,6]. Indeed, Kaškonienė et al. [7] pointed out in their study investigating the antimicrobial and antioxidant activities of natural and fermented bee pollen that fermentation has a positive effect on the biological activities of bee pollen. Still, this positive effect depends on the pollen’s botanical origin and fermentation method.

Bee bread includes approximately 250 components, such as macro- and micronutrients, vitamins, flavonoids, enzymes, amino acids, phenolic compounds, and fatty acids [8]. Bee bread has a higher nutrient content than pollen and has a better digestibility [1,2,4,9]. However, the functional characteristics and chemical composition of bee bread and pollen vary depending on factors such as the plant’s nutritional status, the botanical origin of the pollen, climate, collection, soil, storage, geographical conditions, and processing degree [1,10,11]. Bee bread possesses antioxidant, antifungal, antibacterial, antitumor, antiatherosclerotic, anti-aging, anti-anemic, and liver protective effects [4,11]. Additionally, adding bee pollen and bee bread to foods results in increased nutritional value and antioxidant activity [12].

Ardahan province, which holds significant importance in beekeeping activities in Türkiye, is known for its natural richness. The Ardahan flower honey produced in the province has been registered with a “Geographical Indication Certificate” by the Turkish Patent Institute. However, it is observed that producers do not sufficiently value natural products including bee pollen and bee bread, and awareness in this regard has not been adequately raised. Considering the recent increase in the use of natural products as food supplements, it is crucial to conduct composition analyses of natural bee products available in these regions rich in natural resources. Furthermore, a limited number of studies regarding the chemical composition of bee bread produced in the different areas were encountered during the literature review. In line with these objectives, this study aims to determine the total phenolic, flavonoid, antiradical, organic acid, and sugar contents of bee bread samples collected from Ardahan province in northeastern Türkiye.

## 2. Materials and Methods

### 2.1. Sampling

Bee bread samples were collected from six regions, including Ardahan city center and five districts in the northeastern part of Türkiye’s Eastern Anatolia Region. Its geographical location is 41°6′36″ north, 42°42′12″ east. Samples were collected from six different locations.. The districts of Ardahan are shown (Figure S1). Samples collected from the Center (seven samples), Posof (nine samples), and Hanak (six samples) were obtained from three different beekeepers and different hives. Samples collected from Çıldır (five samples), Damal (five samples), and Göle (five samples) were obtained from two different beekeepers and different hives. The distribution of samples by beekeepers and beehives in the sampled areas is detailed in Table 1. The samples were obtained fresh from beekeepers during the spring season. A total of 37 samples were taken for analysis and they were stored at 4 °C until analyzed.

### 2.2. Analysis of Bioactive Compounds

Bee bread samples (100 mg) were mixed with methanol by vortexing (3000 rpm, 3 min) for total bioactive analyses. The resulting mixtures were further extracted using an ultrasonic bath. The mixtures were centrifuged (10 min, 3500 rpm) and filtered (Whatman 41). For DPPH analysis, methanol was added to dilute the supernatants. These were used directly for the determination of total bioactives. The result for total phenolic content was measured using the Folin–Ciocalteau method [13]. Bee bread, distilled water, and Folin–Ciocalteu reagent were combined in an aliquot comprising 50 μL, 450 μL, and 2.5 mL, respectively. After waiting 5 min in darkness, saturated sodium carbonate (2 mL) was added. Then, the spectrophotometer (SP-UV/VIS-300SRB, Spectrum Instruments, Melbourne, Australia) was used to measure the absorbance at 765 nm. Results were shown as mg gallic acid equivalent/g. The total flavonoid result of the bee bread was carried out using a colorimetric technique according to the study by Zhishen et al. [14]. After preprocessing, the absorbance results were read at 510 nm. Results were shown as mg catechinic values (CE)/g. The bee bread’s antioxidant capacity was assessed using the method based on the scavenger 2,2-diphenyl-1-picrylhydrazyl (DPPH) radical, with some modifications [15]. The results were calculated as DPPH (mg TEAC/g).

### 2.3. Analysis of Phenolic Compounds

The phenolic compounds were analyzed on an Agilent 1260 Infinity chromatograph equipped with a diode array detector (DAD). The chromatography method was reported by Portu et al. [16]. The column used was an Agilent C-18 Age Generix column with dimensions of 250 × 4.6 mm and a packing of 5 µm. The column was fixed at 30 °C and 0.80 mL/min. Solutions A and B were water with 0.1% phosphoric acid and acetonitrile, respectively. In addition, 17% B (0 min), 15% (7 min), 20% (20 min), 24% (25 min), 30% (28 min), 40% (30 min), 50% (32 min), 70% (36 min), and 17% (40 min) were used as gradients. To analyze the fractions of phenolic compounds, 10 µL was injected. Phenolic compounds were identified using standards. The assay was carried out at 280, 320, and 360 nm. The results (μg/g) were calculated as the average of three bee bread sample analyses. 

### 2.4. Analysis of Amino Acids

The amino acid composition was analyzed using the method defined by Bilgin et al. [17], with slight modification. The analysis of the amino acids was carried out using an LC system (Agilent Technologies, Waldbronn, Germany). The MS/MS analyses were performed using an Agilent 6460 triple quadruple LC–MS with an electrospray ionization interface. The amino acid content was analyzed using the JASEM Quantitative Amino Acids Kit protocol (Sem Laboratuvar Cihazları A. Ş., Istanbul, Türkiye). After filtration without acid hydrolysis and dilution, the samples were read in the instrument (mg/100 g). 

### 2.5. Analysis of Organic Acid and Sugar

Samples of bee bread were finely ground using a laboratory mortar. The samples were vortexed (3000 rpm for 3 min) with deionized water (5 mL at 80 °C) and were thawed via incubation in a water bath for 15 min. The samples were centrifuged (10 min, 3500 rpm) and filtered through micro filters with 0.45 μm pore size before being used in the analysis. Following the method proposed by Coelho et al. [18], the organic acids and sugar contents were analyzed using high-performance liquid chromatography (HPLC model 1260 Infinity LC Agilent Technologies, Santa Clara, CA, USA), with minor modifications. A disc syringe filter (0.45 μm) was used to filter samples (500 μL). The ion-exchange column used was an Agilent Hi-Plex H (300 × 7.7 mm). The column compartment temperature was kept at 65 °C, while the RID flow cell was kept at 35 °C. The experiment utilized a flow rate of 0.6 mL/min for a duration of 20 min. The phase was 10.0 mML^−1^ H_2_SO_4_ in ultrapure water. Retention times for each compound were determined using standard solutions. For the determination of citric acid, acetic acid, lactic acid, succinic acid, oxalic acid, tartaric acid, and malic acid detection was conducted in the DAD at 210 nm. RID was used to detect sucrose, glucose, and fructose. Sugars and organic acids are shown as g/100 g and mg/g bee bread sample, respectively.

### 2.6. Statistical Analysis

The assays were conducted in triplicate, and the results were expressed as mean ± standard deviation (SD). Data were analyzed using a one-way analysis of variance (ANOVA) and differences between means were determined using Tukey’s HSD (Honestly Significant Difference) test at a significance level of *p* < 0.05. The statistical analysis was carried out using SPSS 22.0 software (SPSS Inc., Chicago, IL, USA). Principal component analysis (PCA) was performed using JMP (12.2.0 SAS Institute, Cary, NC, USA). The Pearson correlation coefficient was analyzed using OriginPro (version 2017, OriginLab, Northampton, MA, USA).

## 3. Results and Discussion

### 3.1. Total Phenolic Contents and Total Flavonoid Contents

**Figure 1:**
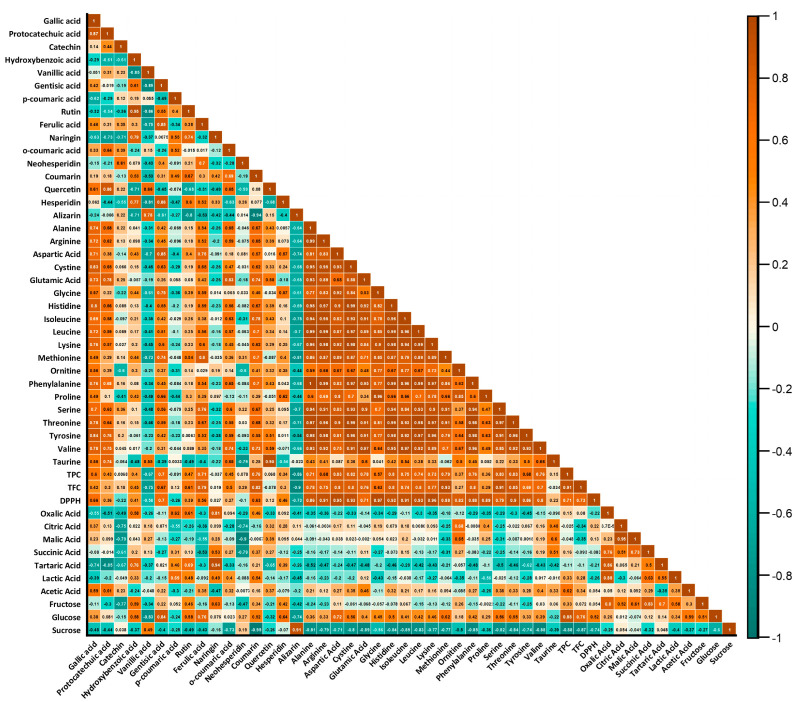
Pearson’s correlation relationship between phenolic compounds, bioactive compounds, amino acids, organic acids, and sugar values of the samples.

Bioactive compounds are secondary metabolites with positive effects on health, including antioxidant, antimicrobial, and anti-inflammatory properties, which have been in the spotlight. These compounds, found in small amounts in foods, exhibit a wide range of chemical structures and functions, with their content varying depending on the plant species [19,20]. The total phenolic contents, total flavonoid contents, and DPPH parameters of bee bread samples are presented in Table 1. The total phenolic values of bee bread samples collected from Ardahan city center and districts ranged from 11.70 to 18.35 mg GAE/g. The highest total phenolic content value was determined in the sample obtained from the Damal district, with a value of 18.35 mg GAE/g, showing a statistically significant difference (*p* < 0.05). When the total phenolic content values of the samples were evaluated statistically, no significant difference was found between apiaries and hives within the same region, except for in the Hanak district (*p* > 0.05). It was determined that total phenols had a high positive correlation with total flavonoids (r = 0.91). A positive correlation was also found between total phenolics and antiradical activity (r = 0.71). Similarly, total flavonoids and antiradical activity exhibited a positive correlation (r = 0.71) (Figure 1). The highest total flavonoid content value in the samples was 2.82 mg QE/g in a sample collected from the Posof district, and a statistically significant difference was found (*p* < 0.05). 

In a study conducted to investigate the total phenolic content, antibacterial, and anti-radical properties of bee bread from Türkiye, it was reported that the total phenolic content value of bee bread was 24.45 g GAE/mg and the DPPH activity was 3.40% [21]. In another study, the total phenolic content, flavonoid content, and antioxidant content of bee bread samples collected from different regions of Anatolia were determined as 11.90–14.77 mg GAE/g, 1.30–6.03 mg CE/g, and 20.03–35.43 mg TEAC/g, respectively (Beykaya et al. [22]). The results we obtained show a higher total phenolic content compared to the findings of this study, while being lower in terms of flavonoid content. Kalaycıoğlu et al. [23] reported that in a study where they evaluated the chemical profile and antioxidant activity of bee bread collected from Anatolia, the total phenolic content of the samples ranged from 13.26 to 32.45 mg GAE/g. They reported that the highest total phenolic compound was found in Trabzon (32.45 mg GAE/g) and Ankara bee bread (28.95 mg GAE/g), while the lowest amount was found in the bee bread sample from Bingöl (13.7 mg GAE/g). In the same study, the total phenolic content in bee bread from Erzurum was reported as 15.0 mg GAE/g. In terms of the total phenolic content, our results are partially consistent with the results of the Eastern Anatolian provinces (Bingöl and Erzurum) in this study. Kowalski and Makarewiz [24] studied the functionality of honey enriched with propolis and bee bread. They mentioned that while the total phenolic content of lemon honey was approximately 36 mg GAE/100 g, the addition of bee bread and propolis to lemon honey resulted in a total phenolic content value of 150 mg GAE/100 g and a DPPH activity of 53 Trolox/100 g. In another study, it was stated that the bee bread (ethanolic extract) had a higher free radical scavenging activity compared to propolis [25]. The most increased antioxidant activity with a value of 93% and a total phenolic content of 394 mg GAE/100 g was found in bee bread samples collected in Northwest Lithuania Bartkiene et al. [26]. In our study, the highest total phenolic content value was 18.35 mg GAE/g, which is quite high compared to the study mentioned. When comparing the results of this study with the results of other studies, it is observed that different results emerge. This difference is thought to be due to differences in vegetation, climate conditions, geographical origin, storage conditions, and collection of bee bread. Additionally, these differences have been associated with the use of different solvents in the extraction procedure [11].

### 3.2. Phenolic Compounds Profile

Bee bread is reported to be rich in phenolic compounds and is, therefore, a functional food [27,28]. The results of the polyphenol analysis of bee bread samples collected from six different districts of Ardahan Province are shown in Table 2. The bee bread samples were analyzed for 19 phenolic compounds. Among these compounds, the highest amount was found in gallic acid polyphenol (19.54–39.97 µg/g). Although high in all districts, the highest amount was determined in the bee bread collected from the Hanak district, followed by Çıldır, Damal, Göle, Center, and Posof, respectively. After gallic acid, the highest amounts found among polyphenols were those of hydroxybenzoic acid (ND-6.78 µg/g) and ferulic acid (0.25–3.25 µg/g). In our study, resveratrol, transcinnamic acid, and flavonoids were not detected. O-coumaric acid was found only in the Posof district. Naringin was found in Posof and Göle, and alizarin in the Centre and Hanak districts. Gallic acid, ferulic acid, protocatechuic acid, vanillic acid, quercetin, and hesperidin were detected in bee bread samples obtained from all districts at varying levels.

The study by Bayram et al. [10] investigated the phenolic compounds of fresh bee bread from five different regions and five hives in Türkiye (Kırklareli/Çağlayıkayık, Bursa/Cumalıkızık, Ankara/Beytepe, Ankara/Kahramankazan, and Rize/Hala); it was noted that the rutin content was relatively high (12,613.49–1225.54 µg/100 g) as a polyphenol in the samples. This value was significantly higher than the rutin polyphenol values obtained in our study on the bee bread from Ardahan province. This suggests that the amount of rutin may vary in bee bread due to differences in flora. The region with the highest gallic acid value (347.37 µg/100 g) was Rize/Hala, which is lower than the highest value obtained in our study, 39.97 µg/100 g in Ardahan/Hanak. While we did not detect resveratrol in our research, it was found in only one region (Rize/Hala) in the same study by Bayram et al. [10]. The absence of catechin, which we could detect in all regions except Göle in our study, was observed in all five regions in the same study [10] and the detection of naringin in two regions in our study, while it was not found in the same study [10], are noteworthy discrepancies. As for the total phenolic compounds, the results of bee bread from Ankara/Kahramankazan were the highest at 10820.84 µg/100 g (45.95 µg/g in our study). The emphasis on differences in quantity and regional variations in bee pollen and bee bread in the same study has also been confirmed by our study. In the same study, it was stated that the predominant botanical sources for bee bread were the *Fabaceae*, *Asteraceae*, Rosaceae, and Plantaginaceae families. In the province of Ardahan, most of the pollen grains come from *Astragalus* spp., *Apiaceae*, and *Brassicaceae*. The pollen grains of *Astragalus* spp., *Brassicaceae*, and *Apiaceae* are of secondary importance, while the pollen of *Lamiaceae* and *Fabaceae* are found in small amounts [29].

Phenolic compounds such as gallic, rosmarinic, p-hydroxybenzoic, luteolin, protocatechuic, myricetin, kaempferol, chlorogenic, caffeic, vanillic, p-coumaric, and quercetin were analyzed in bee bread purchased from Romania. The analysis revealed that the main phenolic compounds present were kaempferol (31.25 mg/L extract), myricetin (3.15 mg/L extract), and luteolin (1.17 mg/L extract). p-hydroxybenzoic acid, protocatechuic acid, gallic acid, chlorogenic acid, and vanillic acid were not detected. Other values (mg/L extract) were reported as follows: caffeic acid 0.10, p-coumaric acid 0.11, rosmarinic acid 0.23, and quercetin 0.06 [28]. In our study, protocatechuic acid, gallic acid, and vanillic acid, which were not detected in this study, were detected in bee bread samples from all regions. Markiewicz-Zukowska et al. [24] reported detecting trace amounts of phenolic compounds in bee bread samples, mainly kaempferol and apigenin. In Lithuania, bee bread obtained from various sources was reported to contain p-coumaric acid, kaempferol, chrysin, and apigenin [30].

To measure the linear relationship, a correlation analysis was used. A positive correlation (r = 0.76) was found between TPC and coumarin in the bee bread samples. Positive correlations were also observed between TFC and coumarin (r = 0.82) and between TFC and ferulic acid (r = 0.79) (Figure 1).

According to the results obtained, phenolic compounds in bee bread may vary in type and amount depending on the pollen source. This variability could pose challenges in product standardization and should be considered, especially when considering the use of bee bread as a raw material in different functional products. Therefore, regional differences must be considered.

### 3.3. Amino Acid Profile

Due to their critical role in human metabolism, including growth, nitrogen balance, protein synthesis, regulation of gene expression, oxidative defenses, gut health, and immune system function, amino acids are considered essential nutrients [31]. Peptides and free amino acids are highly concentrated in bee bread. It has a better composition than many valuable animal protein sources. It contains all the essential amino acids [4]. In our study, 19 free amino acids were identified in bee bread samples. The amino acid content of bee bread is presented in Table 3. It was found that bee bread samples contained all essential amino acids except for tryptophan. Bee bread collected from the Çıldır district was determined to have the highest amino acid content, with statistical significance (*p* < 0.05). Bee bread obtained from the Ardahan city center exhibited the lowest values of all amino acids. The high levels of glutamic acid and proline were of particular note. The high levels of proline and glutamic acid in bee bread are an indication of high-quality pollen. In contrast, low levels of proline may be an indication of low quality and processing [10,32].

In a study conducted by Bayram et al. [10] in Türkiye, the free amino acid content of five different bee bread samples was examined. In addition to the amino acids cystine, glutamic acid, tyrosine, arginine, leucine, aspartic acid, serine, histidine, alanine, valine, glycine, taurine, ornithine, isoleucine, lysine, proline, methionine, phenylalanine, and threonine, they analyzed a total of 42 amino acids, including other amino acids. The study found that proline was dominant in both bee pollen and bee bread samples, followed by L-asparagine in pollen samples and L-aspartic acid in both pollen and bee bread samples [10]. In our study, however, aspartic acid was dominant, followed by pyrroline and glutamic acid. Our study findings were similar to those of the study above. In research conducted by Mohammad et al. [33] in Malaysia, they determined the amino acid profile of bee bread, identifying eight essential amino acids. They reported that arginine was the dominant amino acid with a value of 2.334 g/100 g, followed by phenylalanine with a value of 2.288 g/100 g. In this study, the highest arginine value was found to be 1393.56 mg/100 g, which is lower than that in the mentioned study. Arginine is a vital gluconeogenic amino acid with immunomodulatory effects. Therefore, it is suggested that incorporating bee bread into the daily human diet as a nutraceutical food supplement is necessary [9]. Sommano et al. [34] reported that the dominant essential amino acid types in pollen and honey samples were threonine, phenylalanine, and leucine, with the lowest being proline. In another study conducted in Türkiye, Poyraz et al. [35] found that the highest essential amino acids in all samples were alanine, leucine, and phenylalanine with values of 2.6086 g/100 g, 3.7982 g/100 g, and 14.447 g/100 g, respectively. In our study, the highest phenylalanine, alanine, and leucine values were determined in samples collected from Çıldır, with values of 1219.80 mg/100 g, 1627.32 mg/100 g, and 2251.99 mg/100 g, respectively. Our results are lower compared to the mentioned study. The study findings show significant differences in free amino acid levels among samples collected from different regions. Indeed, bee bread samples collected from various seasons in China showed notable differences in free amino acid levels and other nutritional components [36]. 

### 3.4. Soluble Sugar Profile

**Figure 2:**
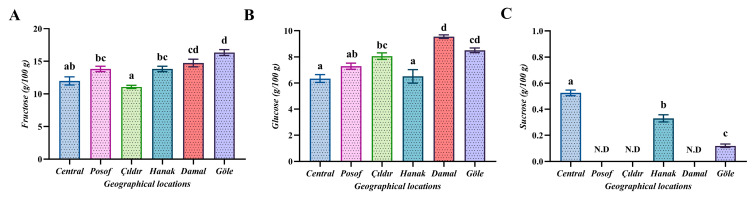
Sugar component analysis results of bee bread (g/100 g). N.D.—not detected. Vertical bars indicate ± standard errors of the mean values (*n* = 3), the same as below. Different letters in the same row indicate significant differences (*p* < 0.05) between the means.

Bee bread is a mixture of pollen particles, honey, and lactic acid bacteria, constituting one of the primary food sources in the hive. It has a high nutritional value and contains easily digestible free sugars. Figure 2 shows the results of the free sugar analysis. Among the sugars analyzed (fructose, glucose, and sucrose), fructose was the highest, with the highest level obtained from the Göle region (16.35 g/100 g). The lowest fructose value was obtained from the Çıldır region (11.07 g/100 g). Regarding glucose levels, the highest value was from the Damal region (9.55 g/100 g), while the lowest was from the Center region (6.34 g/100 g). Sucrose was not detected in the Posof, Çıldır, and Damal regions, whereas it was found in the Göle, Hanak, and Center regions at levels of 0.12, 0.33, and 0.53 g/100 g, respectively. Significant differences were found among the regions according to statistical evaluations. Sugars are the most critical energy source for honey bees. An adult honey bee requires approximately 4 mg of available sugar per day to sustain its vital activities [37]. The carbohydrate composition of bee bread is primarily dependent on the origin of the pollen. The average carbohydrate content of bee bread ranges from 24.40% to 34.80%, with fructose constituting the largest proportion of carbohydrates (57.51%), followed by glucose (42.59%) and maltose (3.37%). The sucrose content remains approximately 0.12% due to the decomposition of sucrose into monosaccharides during the lactic acid fermentation of pollen into bee bread [38]. The average sucrose value of our research was evaluated as 0.16 g/100 g. In their study investigating sugar levels in bee bread, Bakour et al. [11] found levels of glucose (5.7 g/100 g) and fructose (11.8 g/100 g), while our research results yielded an average fructose value of 13.63 g/100 g and an average glucose value of 7.71 g/100 g. In a study by Aylanc et al. [1], fructose (ranging from 21.9 to 23.5 g/100 g) and glucose (ranging from 12.0 to 12.4 g/100 g) were detected, while sucrose was not detected. A study by Kalaycıoğlu et al. [23] found fructose (11.6–32.1 g/100 g) and glucose (6.21–21.5 g/100 g) were the major sugars. The variation in fructose and glucose ratios in bee bread samples can be attributed to regional differences and the composition of pollen. The finding of the highest level of fructose followed by glucose and sucrose in bee bread samples is consistent with the findings of other studies.

### 3.5. Organic Acid Profile

**Figure 3:**
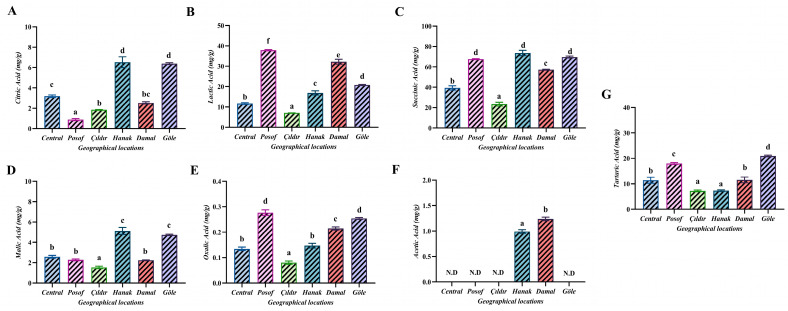
Organic acid analysis results of bee bread. N.D.—not detected. Vertical bars indicate ± standard errors of the mean values (*n* = 3), the same as below. Different letters in the same row indicate significant differences (*p* < 0.05) between the means.

The findings of the organic acid content in bee bread samples are shown in Figure 3. Seven different organic acids (oxalic acid, citric acid, malic acid, succinic acid, tartaric acid, lactic acid, and acetic acid) were analyzed in the samples. The highest value among organic acids was obtained from the Hanak region with 73.63 mg/g of succinic acid. Oxalic acid (0.08 to 0.28 mg/g); citric acid (0.89 to 6.53 mg/g); malic acid (1.54 to 5.12 mg/g); succinic acid (23.42 to 73.63 mg/g); tartaric acid (7.22 to 20.95 mg/g); lactic acid (7.08 to 37.86 mg/g); and acetic acid (0 to 1.24 mg/g) were detected in the mentioned ranges. The amount of acetic acid was not detected in four regions (Center, Posof, Çıldır, and Göle). The differences between the regions are considered significant according to the statistical evaluations conducted. In the Bakour et al. [11] study, only oxalic acid (0.383 g/100 g) was detected as an organic acid in bee bread products. According to our study results, the amount of oxalic acid is, on average, 0.18 mg/g, which is lower compared to the results of other researchers. Kalaycıoğlu et al. [23] detected gluconic acid (43.6–63.2 g/kg) as the predominant organic acid in bee bread samples. Lactic acid (17.6–21.7 g/kg) was determined to be the second highest organic acid. Barene et al. [39] found the level of lactic acid in bee bread to be 3.06–3.20%. According to our findings, lactic acid is, on average, 21.07 mg/g. Based on these results, the high level of lactic acid can be considered an indicator of good fermentation. Especially when compared to pollen, lactic acid levels are approximately six times higher, contributing to increased preservation and inhibiting yeast development in bee bread [3]. 

### 3.6. PCA Analysis

**Figure 4:**
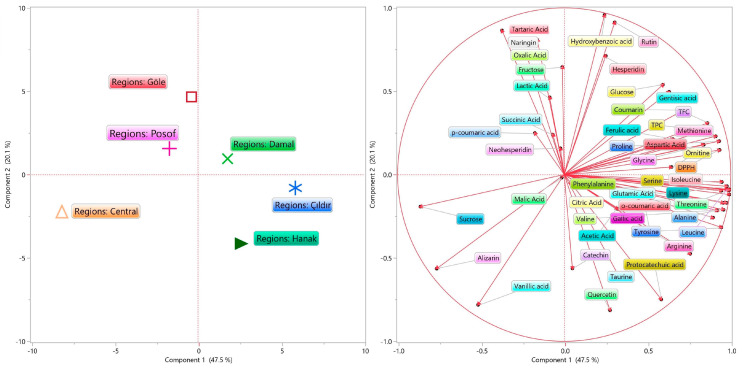
PCA bi-plot of volatile compounds in bee bread samples.

Principal component analysis (PCA) was used to evaluate the differences in total bioactives, free amino acids, phenolic compounds, organic acids, and sugars among the six districts of Ardahan (Figure 4). PCA graphs depict the distribution of samples on two main components. The eigenvector values were obtained for all samples in the score plot; PC1 = 47.5% and PC2 = 20.1%. PCA is suitable for distinguishing bee bread and grouping compounds based on their spatial positions. Damal shows positive loadings on PC1 and PC2. The center shows negative loadings on PC1 and PC2. Çıldır and Hanak show positive loadings on PC1 and negative loadings on PC2. However, Posof and Göle show negative loadings on PC1 and positive loadings on PC2 and are grouped with four organic acid compounds (tartaric, oxalic, lactic, and succinic). The Centre district is positioned with malic acid, sucrose, alirizin, and vanillic acid. Total bioactive compounds (TPC, TFC, and DPPH) are grouped positively with the Damal district, as shown in Figure 4. The most grouping occurred with Çıldır and Hanak districts, especially amino acids that are positioned with these districts. Similarly, PCA has been used in bee bread studies to differentiate regionally, as in our study [10,23]. Bee bread samples can be distinguished using the analysis data obtained with PCA analysis results and the specified factors.

## 4. Conclusions

Bee bread is considered a functional food due to its rich chemical composition and bioactive properties. As the world’s population rapidly increases, food resources decrease and there is a growing demand for natural products. Therefore, there is an especially significant interest in bee products. This study aimed to determine the bioactive compounds, amino acid, sugar, and organic acid profile of bee bread samples from Ardahan, Türkiye. PCA applied to all analysis data obtained for Ardahan bee bread samples showed that the samples were well distinguished. The results obtained are expected to contribute to future studies to further improve and standardize bee bread, which is considered a functional food. In conclusion, more detailed research is needed to determine bee bread’s nutritional, chemical, and bioactive properties in this region.

## Figures and Tables

**Table 1:** Results of total bioactive contents of bee bread samples.

Regions	Beekeeper	Beehives	Analyses		
			TPC (mg GAE/g)	TFC (mg QE/g)	DPPH (mg TEAC/g)
Center	1	1	12.40 ± 0.50 ^a^	2.12 ± 0.03 ^a^	3.13 ± 0.02 ^a^
		2	11.94 ± 0.48 ^a^	2.16 ± 0.05 ^a^	3.15 ± 0.01 ^a^
		3	12.27 ± 0.25 ^a^	2.23 ± 0.12 ^a^	3.13 ± 0.03 ^a^
	2	1	12.25 ± 0.49 ^a^	2.26 ± 0.12 ^a^	3.16 ± 0.12 ^a^
		2	11.70 ± 0.37 ^a^	2.11 ± 0.04 ^a^	3.24 ± 0.03 ^ab^
	3	1	14.01 ± 0.56 ^b^	2.18 ± 0.12 ^a^	3.34 ± 0.03 ^bc^
		2	12.75 ± 0.51 ^ab^	2.08 ± 0.11 ^a^	3.44 ± 0.05 ^c^
Average			12.47 ± 0.75 ^A^	2.16 ± 0.07 ^A^	3.23 ± 0.12 ^A^
Posof	1	1	15.16 ± 0.25 ^a^	2.10 ± 0.11 ^a^	3.38 ± 0.04 ^ab^
		2	15.93 ± 0.66 ^a^	2.82 ± 0.05 ^d^	3.34 ± 0.03 ^ab^
		3	15.21 ± 0.40 ^a^	2.72 ± 0.03 ^cd^	3.31 ± 0.07 ^a^
	2	1	15.23 ± 0.63 ^a^	2.65 ± 0.05 ^cd^	3.46 ± 0.04 ^bc^
		2	14.88 ± 0.51 ^a^	2.58 ± 0.06 ^bcd^	3.41 ± 0.10 ^ab^
		3	15.37 ± 0.13 ^a^	2.71 ± 0.15 ^cd^	3.58 ± 0.04 ^c^
	3	1	15.59 ± 0.61 ^a^	2.44 ± 0.13 ^bc^	3.60 ± 0.05 ^c^
		2	14.64 ± 0.50 ^a^	2.55 ± 0.14 ^bcd^	3.40 ± 0.04 ^ab^
		3	15.72 ± 0.10 ^a^	2.34 ± 0.13 ^ab^	3.40 ± 0.02 ^ab^
Average			15.30 ± 0.40 ^B^	2.55 ± 0.22 ^BCD^	3.43 ± 0.10 ^B^
Çıldır	1	1	17.40 ± 0.54 ^a^	2.75 ± 0.05 ^a^	3.74 ± 0.11 ^a^
		2	15.68 ± 1.15 ^a^	2.65 ± 0.14 ^a^	3.78 ± 0.04 ^a^
		3	16.55 ± 0.77 ^a^	2.71 ± 0.12 ^a^	3.90 ± 0.06 ^b^
	2	1	16.78 ± 0.68 ^a^	2.69 ± 0.09 ^a^	3.69 ± 0.04 ^a^
		2	16.26 ± 1.06 ^a^	2.77 ± 0.15 ^a^	3.81 ± 0.05 ^ab^
Average			16.53 ± 0.64 ^BC^	2.71 ± 0.05 ^D^	3.78 ± 0.08 ^D^
Hanak	1	1	14.83 ± 1.10 ^ab^	2.33 ± 0.09 ^a^	3.53 ± 0.01 ^a^
		2	13.93 ± 1.73 ^a^	2.54 ± 0.14 ^a^	3.60 ± 0.09 ^ab^
	2	1	16.16 ± 0.67 ^ab^	2.44 ± 0.13 ^a^	3.73 ± 0.04 ^b^
		2	16.86 ± 0.36 ^b^	2.47 ± 0.07 ^a^	3.47 ± 0.13 ^a^
	3	1	16.73 ± 0.69 ^b^	2.31 ± 0.12 ^a^	3.64 ± 0.04 ^ab^
		2	16.93 ± 0.21 ^b^	2.53 ± 0.03 ^a^	3.70 ± 0.07 ^b^
Average			15.91 ± 1,25 ^B^	2.44 ± 0,10 ^B^	3.61 ± 0.10 ^C^
Damal	1	1	18.35 ± 0.79 ^a^	2.63 ± 0.15 ^a^	3.51 ± 0.04 ^ab^
		2	17.39 ± 0.21 ^a^	2.62 ± 0.10 ^a^	3.50 ± 0.05 ^ab^
		3	18.03 ± 0.73 ^a^	2.65 ± 0.15 ^a^	3.43 ± 0.02 ^a^
	2	1	18.06 ± 0.63 ^a^	2.69 ± 0.03 ^a^	3.55 ± 0.01 ^b^
		2	17.23 ± 0.17 ^a^	2.80 ± 0.04 ^a^	3.58 ± 0.04 ^b^
Average			17.81 ± 0.48 ^C^	2.68 ± 0.07 ^CD^	3.51 ± 0.06 ^BC^
Göle	1	1	15.57 ± 0.75 ^a^	2.59 ± 0.06 ^a^	3.78 ± 0.06 ^b^
		2	15.79 ± 0.55 ^a^	2.45 ± 0.13 ^a^	3.53 ± 0.05 ^a^
		3	15.52 ± 0.52 ^a^	2.52 ± 0.03 ^a^	3.62 ± 0.02 ^a^
	2	1	16.67 ± 0.58 ^a^	2.34 ± 0.13 ^ab^	3.66 ± 0.15 ^b^
		2	15.18 ± 0.07 ^a^	2.47 ± 0.14 ^a^	3.58 ± 0.03 ^a^
Average			15.75 ± 0.56 ^B^	2.47 ± 0.09 ^BC^	3.63 ± 0.09 ^CD^

TFC: total flavonoid content; TPC: total phenolic content; GAE: gallic acid equivalent; QE: quercetin equivalent; TEAC: Trolox equivalent antioxidant capacity. Lowercase letters indicate statistical differences within hives. Capital letters indicate statistical differences between regions (*p* < 0.05).

**Table 2:** Results of phenolic compounds of bee bread samples.

No.	Phenolic Compounds (μg/g)	Geographical Locations					
		Center	Posof	Çıldır	Hanak	Damal	Göle
1	Gallic acid	22.87 ± 1.54 ^ab^	19.54 ± 0.54 ^a^	35.91 ± 0.99 ^cd^	39.97 ± 1.45 ^d^	34.20 ± 0.48 ^c^	26.39 ± 0.55 ^b^
2	Protocatechuic acid	0.07 ± 0.01 ^b^	0.07 ± 0.00 ^b^	0.12 ± 0.00 ^c^	0.17 ± 0.01 ^d^	0.12 ± 0.00 ^c^	0.04 ± 0.00 ^a^
3	Catechin	0.65 ± 0.04 ^bc^	0.58 ± 0.02 ^b^	0.72 ± 0.02 ^c^	0.45 ± 0.02 ^a^	0.68 ± 0.01 ^c^	N.D
4	Hydroxybenzoic acid	N.D	3.75 ± 0.10 ^b^	3.15 ± 0.09 ^a^	N.D	3.00 ± 0.04 ^a^	6.78 ± 0.14 ^c^
5	Vanillic acid	0.45 ± 0.03 ^c^	0.29 ± 0.01 ^b^	0.16 ± 0.00 ^a^	0.45 ± 0.02 ^c^	0.16 ± 0.00 ^a^	0.13 ± 0.00 ^a^
6	Gentisic acid	N.D	N.D	0.34 ± 0.01 ^c^	0.05 ± 0.00 ^a^	0.32 ± 0.00 ^bc^	0.32 ± 0.01 ^b^
7	*p*-coumaric acid	N.D	0.02 ± 0.00	N.D	N.D	N.D	N.D
8	Rutin	N.D	0.53 ± 0.01 ^b^	0.40 ± 0.01 ^a^	N.D	0.38 ± 0.01 ^a^	0.60 ± 0.01 ^c^
9	Ferulic acid	0.25 ± 0.02 ^ab^	0.42 ± 0.01 ^b^	3.25 ± 0.09 ^d^	0.24 ± 0.01 ^a^	3.09 ± 0.04 ^d^	1.12 ± 0.02 ^c^
10	Naringin	N.D	0.11 ± 0.00 ^a^	N.D	N.D	N.D	0.13 ± 0.00 ^b^
11	*o*-coumaric acid	N.D	0.29 ± 0.01 ^b^	0.18 ± 0.00 ^a^	0.28 ± 0.01 ^b^	0.17 ± 0.00 ^a^	N.D
12	Neohesperidin	0.78 ± 0.05 ^c^	0.52 ± 0.01 ^b^	0.95 ± 0.03 ^d^	N.D	0.91 ± 0.01 ^d^	0.37 ± 0.01 ^a^
13	Coumarin	N.D	0.28 ± 0.01 ^b^	0.22 ± 0.01 ^a^	0.20 ± 0.01 ^a^	0.21 ± 0.00 ^a^	0.20 ± 0.00 ^a^
14	Resveratrol	N.D	N.D	N.D	N.D	N.D	N.D
15	Quercetin	0.19 ± 0.01 ^b^	0.20 ± 0.01 ^b^	0.20 ± 0.01 ^b^	0.36 ± 0.01 ^c^	0.19 ± 0.00 ^b^	0.13 ± 0.00 ^a^
16	trans-cinnamic acid	N.D	N.D	N.D	N.D	N.D	N.D
17	Hesperidin	0.14 ± 0.01 ^b^	0.04 ± 0.00 ^a^	0.35 ± 0.01 ^c^	0.04 ± 0.00 ^a^	0.33 ± 0.00 ^c^	0.60 ± 0.01 ^d^
18	Alizarin	0.13 ± 0.01 ^b^	N.D	N.D	0.04 ± 0.00 ^a^	N.D	N.D
19	Flavon	N.D	N.D	N.D	N.D	N.D	N.D
Total		25.53 ± 1.72 ^a^	26.65 ± 0.74 ^a^	45.95 ± 1.26 ^c^	42.26 ± 1.50 ^c^	43.75 ± 0.61 ^c^	36.81 ± 0.77 ^b^

Results are presented as mean ± standard deviation (*n* = 3). Values with different letters within the line are significantly different (*p* < 0.05). N.D.—not detected.

**Table 3:** Results of free amino acid content of bee bread samples.

No.	Amino Acid Content (mg/100 g)	Regions					
		Center	Posof	Çıldır	Hanak	Damal	Göle
1	Alanine	678.57 ± 1.89 ^a^	1128.61 ± 0.74 ^c^	1627.32 ± 0.28 ^f^	1414.37 ± 37.25 ^d^	1191.16 ± 1.19 ^d^	1007.69 ± 0.32 ^b^
2	Arginine	519.25 ± 17.02 ^a^	907.02 ± 10.87 ^ab^	1393.56 ± 24.62 ^e^	1174.51 ± 3.95 ^d^	927.57 ± 0.33 ^c^	869.62 ± 13.50 ^b^
3	Aspartic Acid	1785.48 ± 8.37 ^a^	2333.18 ± 1.26 ^b^	3884.55 ± 8.10 ^e^	3091.92 ± 21.70 ^c^	3319.64 ± 1.75 ^d^	3315.97 ± 12.65 ^d^
4	Cystine	66.22 ± 0.49 ^a^	82.87 ± 0.05 ^b^	114.71 ± 0.02 ^f^	104.69 ± 0.25 ^e^	99.32 ± 0.64 ^d^	90.95 ± 0.07 ^c^
5	Glutamic Acid	1439.47 ± 5.91 ^a^	2632.17 ± 14.03 ^c^	3094.58 ± 29.25 ^e^	3247.31 ± 8.24 ^f^	2841.48 ± 4.74 ^d^	2051.33 ± 19.43 ^b^
6	Glycine	576.79 ± 4.73 ^a^	707.34 ± 2.08 ^b^	1270.73 ± 1.02 ^f^	916.32 ± 9.06 ^d^	815.19 ± 0.06 ^c^	1052.32 ± 4.11 ^e^
7	Histidine	294.98 ± 0.76 ^a^	439.44 ± 1.53 ^b^	632.17 ± 0.08 ^f^	575.03 ± 2.32 ^e^	516.46 ± 0.35 ^d^	460.68 ± 2.76 ^c^
8	Isoleucine	286.18 ± 1.72 ^a^	643.85 ± 2.24 ^b^	854.51 ± 0.43 ^d^	849.68 ± 1.64 ^d^	641.52 ± 0.87 ^b^	660.03 ± 4.40 ^c^
9	Leucine	819.94 ± 0.01 ^a^	1487.52 ± 1.66 ^b^	2251.99 ± 0.67 ^e^	1889.85 ± 13.03 ^d^	1591.15 ± 1.40 ^c^	1490.54 ± 15.54 ^b^
10	Lysine	716.94 ± 2.34 ^a^	1037.67 ± 11.34 ^b^	1625.74 ± 24.02 ^e^	1368.68 ± 1.19 ^d^	1189.34 ± 31.21 ^c^	1176.17 ± 2.05 ^c^
11	Methionine	213.85 ± 1.29 ^a^	359.89 ± 1.05 ^c^	528.42 ± 2.15 ^f^	341.07 ± 0.80 ^b^	406.45 ± 1.74 ^e^	369.96 ± 0.44 ^d^
12	Ornitine	1.47 ± 0.11 ^a^	3.14 ± 0.84 ^ab^	6.55 ± 1.76 ^bc^	8.04 ± 0.80 ^c^	2.71 ± 0.37 ^ab^	7.78 ± 1.62 ^c^
13	Phenylalanine	485.90 ± 1.05 ^a^	852.78 ± 0.60 ^c^	1219.80 ± 1.66 ^f^	1093.88 ± 0.99 ^e^	935.99 ± 2.05 ^d^	792.20 ± 0.96 ^b^
14	Proline	2193.09 ± 2.02 ^a^	2219.95 ± 8.75 ^a^	3453.15 ± 11.79 ^e^	2836.74 ± 7.13 ^c^	2343.86 ± 3.69 ^b^	3271.13 ± 15.82 ^d^
15	Serine	726.90 ± 1.92 ^a^	1052.95 ± 2.48 ^c^	1427.24 ± 0.28 ^f^	1160.51 ± 17.07 ^d^	1252.18 ± 1.84 ^e^	935.49 ± 1.20 ^b^
16	Threonine	519.68 ± 0.42 ^a^	789.28 ± 0.30 ^b^	1150.4 ± 0.60 ^f^	988.25 ± 1.60 ^e^	952.81 ± 5.24 ^d^	800.93 ± 3.73 ^c^
17	Tyrosine	208.89 ± 1.68 ^a^	380.83 ± 0.16 ^b^	723.19 ± 1.43 ^e^	648.72 ± 4.26 ^d^	482.76 ± 0.03 ^c^	373.19 ± 3.01 ^b^
18	Valine	414.83 ± 1.74 ^a^	799.03 ± 6.44 ^c^	1003.35 ± 2.27 ^f^	1104.71 ± 13.84 ^e^	865.74 ± 0.61 ^d^	734.77 ± 1.93 ^b^
19	Taurine	49.67 ± 0.03 ^a^	50.81 ± 1.28 ^ab^	50.13 ± 1.87 ^ab^	54.21 ± 0.44 ^b^	50.10 ± 0.55 ^ab^	49.87 ± 1.15 ^a^
Total		11,998.11 ± 33.04 ^a^	17,908.32 ± 26.04 ^b^	26,312.08 ± 33.14 ^f^	22,868.49 ± 40.55 ^e^	20,425.42 ± 35.88 ^d^	19,510.6 ± 60.01 ^c^

Results are presented as mean ± standard deviation (*n* = 3). Values with the different letters within the line are significantly different (*p* < 0.05).

## Data Availability

The original contributions presented in the study are included in the article/Supplementary Material, further inquiries can be directed to the corresponding authors.

## Supplementary Materials

The following supporting information can be downloaded at: https://www.mdpi.com/article/10.3390/foods13050795/s1, Figure S1. Districts of Ardahan.
