# Supplementary figures and images for: Bee Bread as a Functional Product: Phenolic Compounds, Amino Acid, Sugar, and Organic Acid Profiles
Source: Foods. 2024 Mar 4;13(5):795. doi: 10.3390/foods13050795 (PMC10931289; doi:10.3390/foods13050795)

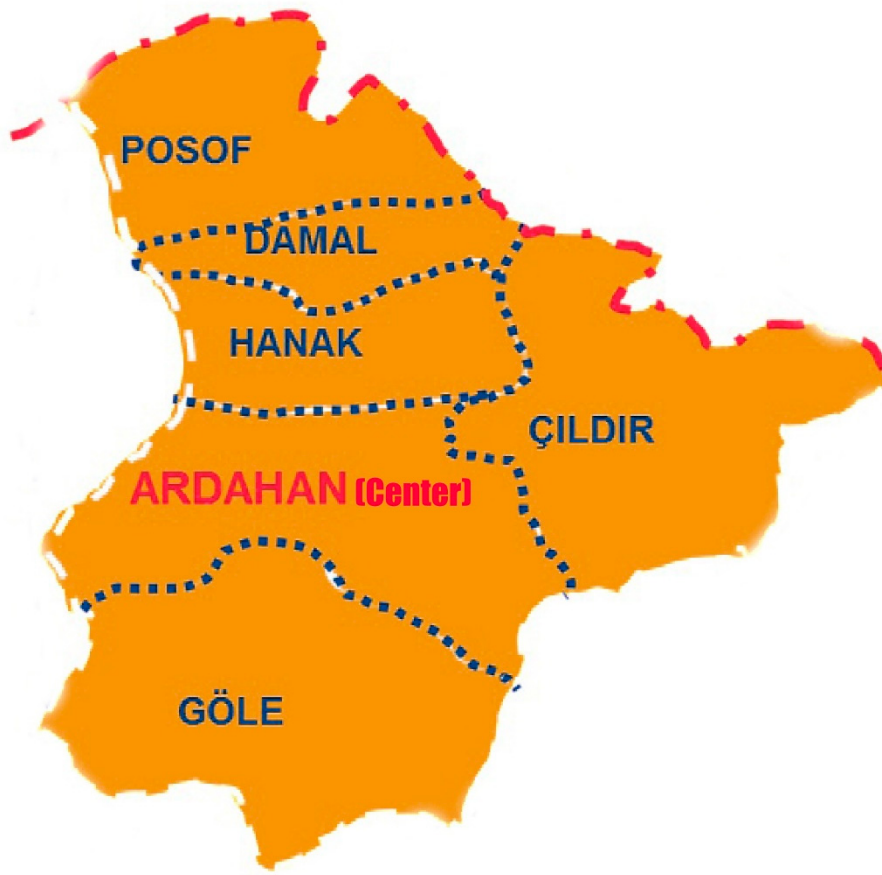

**Figure S1.** Districts of Ardahan.

Supplement: Supplementary file 1 [file foods-13-00795-s001.zip › foods-2894235-supplementary.pdf]
